# Comparison of two tDCS protocols on pain and EEG alpha-2 oscillations in women with fibromyalgia

**DOI:** 10.1038/s41598-020-75861-5

**Published:** 2020-11-03

**Authors:** Géssika Araújo de Melo, Eliane Araújo de Oliveira, Suellen Mary Marinho dos Santos Andrade, Bernardino Fernández-Calvo, Nelson Torro

**Affiliations:** 1 Department of Psychology, Federal University of Paraiba, João Pessoa, 58051-900 Brazil; 2 Department of Physiotherapy, Federal University of Paraiba, João Pessoa, 58051-900 Brazil; 3 Department of Psychology, University of Córdoba, 14071 Córdoba, Spain; 4 Maimonides Biomedical Research Institute of Cordoba (IMIBIC), Reina Sofia University Hospital, University of Cordoba, Córdoba, Spain

**Keywords:** Neuroscience, Diseases, Health care

## Abstract

Transcranial Direct Current Stimulation (tDCS) has been used as an alternative treatment for pain reduction in fibromyalgia. In this study, in addition to behavioral measures, we analyzed oscillations in alpha 2 frequency band in the frontal, occipital, and parietal regions, in response to the application of two neuromodulation protocols in fibromyalgia. The study was a randomized, double-blind, placebo-controlled clinical trial with 31 women diagnosed with fibromyalgia. The participants were allocated to three groups with the anodic stimulation applied on the left motor cortex: Group 1, for five consecutive days; Group 2, for 10 consecutive days; and Group 3, sham stimulation for five consecutive days. Statistical analysis showed a reduction in pain intensity after treatment for groups in general [F (1.28) = 8.02; *p* = 0.008; *η*^2^ = 0.223], in addition to a reduction in alpha 2 in the frontal (*p* = 0.039; *d* = 0.384) and parietal (*p* = 0.021; *d* = 0.520) regions after the treatment on five consecutive days. We conclude that neuromodulation protocols produced similar effects on pain reduction, but differed with respect to the changes in the alpha 2 frequency band in the frontal and parietal regions.

## Introduction

Fibromyalgia (FM) is a clinical condition characterized by the presence of generalized and disabling chronic pain, and may involve symptoms of depression and anxiety^1^. Its diagnosis is based on exclusively clinical criteria, and there are no complementary tests that contribute to its identification.

A challenge in relation to FM is the therapeutic possibilities, as the drugs available for the treatment of chronic pain have provided only modest relief for these patients^2^. Thus, the technique of Transcranial Direct Current Stimulation (tDCS) has shown promising results in the treatment of chronic pain in this population^3^. The application of tDCS has been widely studied in other pain syndromes^2,4,5^. However, the current evidence is still very limited in relation to the ideal treatment protocol, such as frequency and duration of stimulation^3,4^, especially in relation to FM.

The application of anodic tDCS over M1 has shown positive results in pain levels^2^ and that repeated stimulation causes superior results in analgesia in individuals with FM^6^. The effectiveness of tDCS for five days was found by Fagerlund, Hansen and Aslaksen^7^, who found that stimulation was able to promote pain relief without providing serious adverse effects when testing the effect of tDCS in participants with FM in a hospital environment. Mendonca et al.^8^, with a similar protocol, found that the intervention with tDCS provided a reduction in pain and anxiety in individuals with FM. On the other hand, Valle et al.^9^ used a 10-day consecutive tDCS protocol, and also achieved improvement in pain intensity in FM and long-term clinical benefits with stimulation in M1. Research capable of comparing different protocols would help to clarify the best parameters and duration of treatment to be used for pain in FM, enabling the consolidation of a therapeutic protocol.

In the present study, we compared the two more frequently used protocols for the pain treatment of FM, in which the anodic stimulation is applied to M1 for five and ten consecutive days. An advantage of comparing protocols with different durations is to identify the tDCS protocol that produces satisfactory responses with a smaller number of sessions, consequently, minimizing the occurrence of adverse effects and reducing the total time and costs of the therapeutic intervention.

In addition to pain measures, we analyzed the cortical electrical activity associated to the tDCS stimulation, which had not yet been investigated with these protocols in this population. Studies providing an electrophysiological measure of response to the tDCS treatment may help to provide another data in addition to the behavioral one. In this sense, the electroencephalogram (EEG) stands out as a tool for monitoring response to treatment^10,11^.

Although there is disagreement in the literature, in general, the analysis of chronic pain in FM through the EEG shows amplitude of the altered alpha wave, being more studied in the frontal, parietal and occipital regions^10,12^. Alpha is commonly related to the state of relaxation^13^. Villafaina et al.^14^ observed that individuals with FM showed a decrease in the alpha 2 power range in the resting condition, suggesting that chronic pain in these patients modulates this frequency range throughout time. For this reason, the alpha 2 frequency band was the subject of our study. Theoretically, the manipulation of the amplitude of the frequency bands could be associated with behavioral changes^13^. Therefore, the scarcity of studies that offer behavioral and physiological measures of response to treatment with tDCS underscore the importance of this study.

In the present research, we compared two tDCS protocols for pain and their electroencephalographic correlations at rest in women with FM in the frontal, parietal and occipital regions. Our general hypothesis was that different tDCS protocols would lead to a decreased in pain, and differentially modulate the cortical electrical activity in women with fibromyalgia.

## Results

The average age of the participants was 44.81 years old (SD = 8.8) and the average level of pain reported was 6.66 (SD = 1.70) in the VAS. The mean time of diagnosis of FM was 6.60 years (SD = 5.38) and there was no difference between this measure in the groups [F (2, 28) = 2.84; *p* = 0.075]. In addition, 77.40% reported medication use, 25.8% practiced physical activity and 32.30% did psychotherapy. These measures also did not differ between groups [drugs (χ^2^_(2)_ = 0.26, *p* = 0.878); physical activity (χ^2^_(2)_ = 1.67, *p* = 0.404); psychotherapy (χ^2^_(2)_ = 0.59, *p* = 0.747)]. The participants did not report any complaints of adverse effects after the stimulation sessions, nor did they report any neurological complaints or comorbidities, assessed by the CIRS. Also, the groups did not differ from each other before treatment in terms of pain level [F (2,28) = 2.08 *p* = 0.143], anxiety levels [F (2,28) = 0.57 *p* = 0.575], depression [F (2, 28) = 0.36 *p* = 0.699] and cognitive status [F (1, 28) = 2.91, *p* = 0.071].

There was an effect of the time factor on the pain variable [F (1, 28) = 8.02; *p* = 0.008; *η*^2^ = 0.223], with a reduction in pain levels in general after treatment. However, there were no statistically significant differences for the group factor [F (2, 28) = 0.24; *p* = 0.792; *η*^2^ = 0.017], and no interaction between time and group factors [F (2, 28) = 0.90; *p* = 0.417; *η*^2^ = 0.061].

Regarding the electrophysiological variables, there was an effect of the interaction between time and group on the frontal alpha 2 [F (1 28) = 3.62; *p* = 0.040; *η*^2^ = 0.261] and parietal alpha 2 [F (1, 28) = 4.95; *p* = 0.014; *η*^2^ = 0.261] variables, and the participants who received stimulation for five consecutive days showed a significant reduction in the mean alpha spectral power post-intervention in the frontal (*p* = 0.039; *d* = 0.384 ) and parietal (*p* = 0.021; *d* = 0.520) regions. For the alpha 2 in the occipital region, there was no interaction effect [F (1, 28) = 2,452; *p* = 0.104; *η*^2^ = 0.149]. Means groups for electrophysiological and pain variables are shown in Table 1.

**Table 1:** Comparison between the three groups of participants before and after the tDCS treatment on the outcome measures.

Variable	5-days		10-days		Sham		ANOVA		
	Before	After	Before	Aafter	Before	After	Groups (5-days vs 10-days vs sham)	Times (before vs after treatment)	Interaction between groups and times
Pain	7.00 (1.73)	5.05 (2.49)	5.72 (1.56)	5.22 (3.35)	7.09 (1.61)	4.82 (3.34)	p = 0.792 η^2^ = 0.017	p = 0.008 η^2^ = 0.223	p = 0.417 η^2^ = 0.061
Frontal alpha 2	1.07 (3.30)	− 0.24* (3.51)	1.67 (2.41)	1.80 (3.07)	1.95 (2.79)	2.92 (3.62)	p = 0.297 η^2^ = 0.083	p = 0.854 η^2^ = 0.001	p = 0.040* η^2^ = 0.261
Parietal alpha 2	3.77 (3.14)	2.00* (3.63)	5.31 (2.79)	5.76 (4.07)	2.82 (4.74)	4.19 (5.10)	p = 0.302 η^2^ = 0.082	p = 0.964 η^2^ = 0.001	p = 0.014* η^2^ = 0.261
Occipital alpha 2	6.15 (2.99)	4.35 (3.31)	5.80 (3.57)	5.99 (4.27)	6.20 (5.23)	7.20 (4.86)	p = 0.680 η^2^ = 0.027	p = 0.716 η^2^ = 0.005	p = 0.104 η^2^ = 0.149

Values are presented as a function of means and standard deviations. For ANOVAs, *p* and *η*^2^ values are presented for the main factors (times and groups) and for the interaction between them. *Post hoc analyzes indicated a decrease in spectral power in frontal and parietal regions after the application of the 5-days tDCS protocol. Level of significance considered was of p < 0.05. *SD* Standard deviation; *η*^2^: Partial eta-squared as a measure of size effect.

## Discussion

In the present study, we compared two tDCS protocols for pain and their associated electroencephalographic changes in the frontal, parietal and occipital regions in women with FM. We found no difference between the tDCS protocols applied to M1 for 5 days and 10 days on the reported pain. These results are in line with other studies that found no statistically significant difference between active tDCS and sham on pain levels in FM^15^, but disagree with the findings of Fagerlund et al.^7^ and Valle et al.^9^, who reported pain symptom alleviation after the tDCS protocols applied to M1 for 5 days and 10 days, respectively, when compared to placebo.

It is important to highlight that the optimal parameters of the tDCS administration still need to be defined^4^. Considering that studies vary in different aspects, information about the optimal parameters cannot be easily obtained from a comparison between these distinct studies^16^. Previous findings demonstrate limits of the tDCS technique in inducing changes in the cortical excitability^17–19^. Therefore, it is possible that our results indicate a ceiling effect of the cortical changes, so that it would be necessary to test longer protocols to overcome an eventual plateau of brain responses induced by a 5 to 10-days protocol.

In the present study, we found that tDCS modulated cortical electrical activity, with a decrease in alpha 2 spectral power in the parietal and frontal regions after the treatment with the 5-days stimulation protocol. Similarly, Spitoni et al.^20^ found changes in alpha activity in the frontal and parietal regions, but not in the occipital region. The increased alpha amplitude is commonly associated with cortical deactivation and inhibition^21^. Anodic tDCS is commonly associated with increased cortical excitability, so a decline in alpha amplitude is expected after anodic stimulation^20^ as observed in our study for the 5-days stimulation group. In the other two groups, we observed an increase in alpha 2 activity, but below a statistically significant level. Perhaps, this phenomenon corresponds to the effects of tDCS on inhibitory neurons, which could increase the alpha amplitude after stimulation^20^. Considering that tDCS is capable of promoting changes in neuronal excitability^3^, the neuromodulation of the cortical activity may be associated with the decrease in pain.

At the same time, the present results indicate a placebo response to the application of the sham protocol. Other tDCS studies reported similar findings^22^. Placebo analgesic effects can be brought about by the expectation of symptom improvement^17–19^. Moreover, a recent meta-analysis of randomized controlled trials showed that the placebo treatment is clinically effective in reducing pain in FM, and stronger in people with greater pain intensity^23^. The magnitude of the placebo effect in FM may also be influenced by other factors as well as age, gender, disease duration, and expected strength of treatment^23^. This influence of expectations in the tDCS outcomes has been reported in other studies^24,25^. Here, another possible explanation for the placebo effect is the parallel design of the study. A recent metanalysis of non-invasive brain stimulation work showed a significant effect of the placebo in parallel designs, but not in crossover studies^26^. Therefore, further studies may compare tDCS protocols using a parallel design, with the inclusion of control without treatment (waiting list). Likewise, the brain's ability to modify activity in some of its specific structures in response to analgesia due to the placebo effect^27,28^ may justify the fact that there was analgesia associated with an increase in mean potency alpha2 spectral in the parietal cortex in the sham group.

The results reported here must be considered in light of some limitations. First, the high variability in the diagnosis time, and, consequently, the time living with pain may have influenced the results. To minimize this bias, it was ensured that the groups would be homogeneous in relation to the time of diagnosis. Second, the electric field distribution of the tDCS is influenced by the anatomical distribution of the head tissue. Hence, there is only a limited control on the resulting current distribution in the brain^29^. Inter-individual variations of the generated fields are likely a key factor that contributes to the observed physiological and behavioral variability, which may explain the differences between groups with regard to the cortical changes. A computational model may provide information on the distribution of tDCS current in the brain as a function of anatomical factors. However, this method is costly and requires the participants to undergo an MRI, previously to the tDCS application^30^. Nevertheless, future studies may perform the computational modeling of the current in order to control the variability among the individuals with FM.

In conclusion, we found that the tDCS protocols, with the anodic stimulation on M1, for five and 10 consecutive days, as well as the sham protocol, produced similar results in the reduction of pain in women with FM. Nevertheless, the two tDCS protocols modulated alpha 2 cortical electrical activity in the frontal and parietal cortex in different ways. The alpha band is normally associated with a relaxed, passive, and defocused attention state, therefore the modulation of alpha 2 may be related to behavioral changes in women with FM^13^. Future studies may analyze the effects of longer-term tDCS protocols on pain and brain activity modulation.

## Methods

The present study was a longitudinal, randomized, double-blind, placebo-controlled clinical trial, developed for women with fibromyalgia. The project was approved by the Research Ethics Committee of the Health Sciences Center of the Federal University under CAAE: 39796914.5.0000.5188. Written authorization was collected for the participation of each volunteer in the research, through the Informed Consent Form. Participants' autonomy and anonymity was guaranteed, ensuring their privacy regarding confidential data, as regulated by Resolution 466/2012 of the National Health Council. The ethical principles expressed in the Declaration of Helsinki were respected. Our clinical trial was registered on the Clinical Trials platform on 12/28/2017 and is available for public access on the website clinicaltrials.gov through the protocol NCT03384888. It was also registered on the ReBec platform (ensaiosclinicos.gov.br) with protocol RBR-5XBWJK on 06/24/2020. The criteria of the Consolidated standards for test reports—CONSORT were followed.

### Sample

The sample was not probabilistic, and comprised 31 volunteers, aged between 27 and 58 years old, who met the following inclusion criteria: (1) having a diagnosis of fibromyalgia, according to the criteria of the American College of Rheumatology; (2) having been diagnosed at least three months ago; (3) be female; (4) be in the age group between 25 and 60 years old; and (5) signing the Informed Consent Form. It was excluded women with a score below 24 on the Mini Mental State Examination (MMSE); metal implants located on the head, cochlear implants and cardiac pacemaker; illiterate; pregnant women; history of seizure; and severe depression, with a score greater than 35 in the Beck Depression Inventory (BDI).

**Figure 1:**
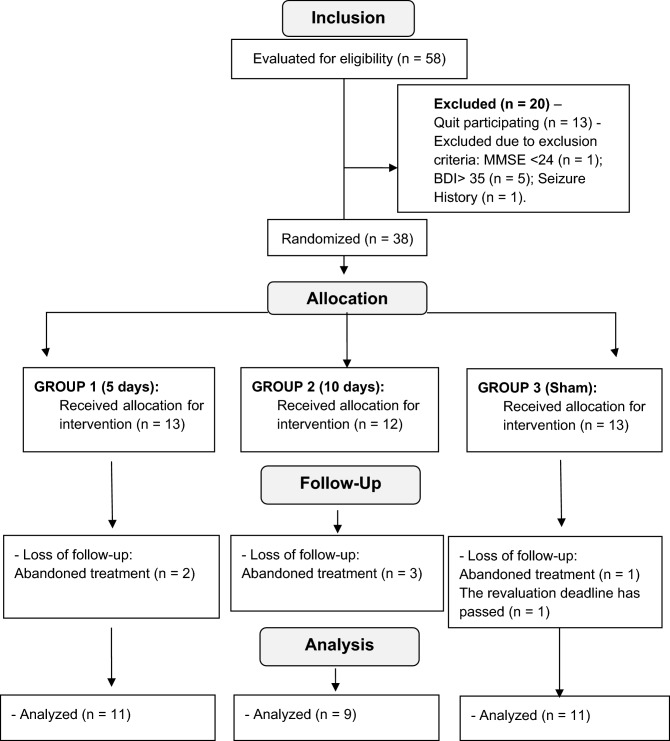
Flowchart of CONSORT showing the outline of the study groups and the respective sample losses.

Participants were randomly assigned to three groups: 11 women in Group 1, with anodic stimulation on the left M1 and cathodic stimulation on the right supraorbital region on five consecutive days; nine women in Group 2, with anodic stimulation on the left M1 and cathodic stimulation on the right supraorbital region on 10 consecutive days (excluding the weekend); and 11 women in Group 3 (sham), with simulated type stimulation, following the protocol of Group 1. All volunteers were reevaluated within seven days after the end of care to ensure the measurement of the effects resulting from the application of the current. Prior to the start of consultations, training was conducted with the examiners to minimize random errors and between researchers. The training ended after the standardization of the process was ensured. The flow of participants in the study is described in Fig. 1. The evaluations and neuromodulation sessions were carried out in the Neuroscience Laboratory, individually.

### Randomization and blinding

The participants were randomly distributed, by one of the researchers, with block exchange at the rate of 1:1:1 using the online randomization program (www.random.org). After randomization, the generated codes were placed in sequential numbered, opaque envelopes and sealed in order to hide the allocation. These envelopes were delivered to the researcher responsible for neurostimulation the day before the start of the sessions. The outcome evaluators and patients were blinded to the type of stimulation applied and the person responsible for neurostimulation blinded to the performance achieved by patients in the evaluations.

### Friction and adhesion

As a friction, it was considered the fault in two sessions or a single fault without replacement. In addition to the insertion of medication for continuous use after the initial evaluation. In order to facilitate the participants' adherence to the study, flexible hours for appointments were organized. It was also allowed to miss a day of attendance, being replaced at the end of the sessions, in addition to making periodic calls in order to maintain contact and avoid evasion from the study.

### Outcome assessment tools

The instruments used for data collection were: the Sociodemographic and Clinical Questionnaire to characterize the sample; the Cumulative Illness Rating Scale (CIRS)^31^, for the analysis of existing comorbidities; the Visual Analogue Scale (VAS)^32^, to check the level of pain at the time of the evaluation; the Mini Mental State Examination (MMSE)^33^, to assess the participants' cognitive status and serve as an exclusion criterion from the study; the Beck Depression Inventory^34^, to exclude participants with severe depression; the Beck Anxiety Inventory (BAI)^35^, to verify that all were homogeneous in terms of anxiety level; and the electroencephalogram, to assess cortical electrical activity. The study phases are shown in Table 2.

**Table 2:** Demonstrative schedule of the study phases—CONSORT 2010.

Time	Recruitment	Allocation	Post-allocation step services		Conclusion
	− t_1_	0	t_1_ 1ª week	t_2_ 2ª week	t_3_
**Recruitment**					
Screening selection	x				
Informed consent	x				
Other procedures	x				
Allocation		x			
**Interventions**					
Group 1 intervention			x		
Group 2 Intervention			x	x	
Group 3 Intervention			x		
Assessments:	x				
Revaluations					x

The clinical trial was conducted between 2018 and 2019.

### Evaluation protocol with electroencephalogram

The data collection process with the EEG was made from 32 electrodes placed on the scalp through an adjustable cap, following the EEG International System 10–20, with impedance below 20kΩ^36^. The amplifier used was the ActiChamp, with a sampling rate of 500 Hz. During the collection, the participants sat comfortably in a chair and were instructed to avoid excessive body and eye movements, in addition to relaxing the mandibular musculature and avoiding muscle contractions in the face region, to decrease the presence of artifacts in the records during the acquisition of the data. Data was collected at rest, 6 min with the participant with eyes open and 6 min with eyes closed^37^. The time was divided into 2 min separated by small intervals and repeated three times, ending in 12 min for the acquisition of data^38^.

### Neuromodulation protocol

The consultations with the tDCS were performed in an individual session and the electrodes were placed in C3, which corresponds to the region of the primary motor cortex (M1), according to SI 10–20 of the EEG^39^. The protocol used was 20 min of stimulation per day, the first group was stimulated for five consecutive days, and the second group for two weeks (excluding weekends), totaling 10 sessions. The protocol for sham stimulation was identical to the first group, but the device was turned off 30 s after the start of stimulation, so as not to induce clinical effects. The TCT research equipment was used, with electrodes wrapped in 5 × 7 cm sponges, moistened with saline (NaCl 0.9%). The applied current was 2 mA, the current density being equivalent to 0.05 A / m2. At the end of each session, the participants were asked about the experience of adverse effects, in order to monitor the safety of applying the current.

### Analysis of electroencephalographic data

The power spectra of the frequency bands for the electrodes F3, F4, P3, P4, O1 and O2 were analyzed, each representing a cortical area—left and right frontal, left and right parietal, left and right occipital—respectively^40^, according to the EEG International System 10–20. The analyzes were performed using EEGLAB, a MATLAB toolbox. In the pre-processing, the data filtering was done using the 0.5 Hz high pass and 30 Hz low pass filter. The average of the electrodes was used as a reference, in order to remove possible spatial biases^10,36^ and, in sequence, the Multiple Artifact Rejection Algorithm—MARA^41^ for removing artifacts. Only the data corresponding to the participants with their eyes closed were processed^37^.

### Statistical analysis

Statistical analyses were carried out using the Software Statistical Package for the Social Sciences (SPSS) version 22.0 for Windows. First, descriptive analyses were performed, using measures of central tendency and dispersion, to characterize the sample. For inferential analyses, initially the Shapiro–Wilk test was used, which indicated that the data had a normal distribution. The chi-square test was used to compare groups with respect to the practical variables of physical activity, psychotherapy and medication use. The one-way ANOVA test was performed to verify homogeneity between groups before the start of treatment. For pre- and post-treatment evaluation, the ANOVA factorial statistical test with mixed design was used, referring to the three groups (Group 1, Group 2 and Sham) x two times (pre and post treatment). The level of significance considered was *p* < 0.05. For peer comparison, the Bonferroni-Sidak post hoc test was used. Finally, we used the effect size calculation from the partial eta squared for each variable within each group, with values of 01, 0.06, and > 0.14 reflecting small, medium, and large effects, respectively and Cohen´s d for comparisons between pairs, with values of 0.20, 0.50, and 0.80 reflecting small, medium, and large effects, respectively^42^.
